# Mobile Application-Based Support for Periodontal Treatment Improves Clinical, Cognitive, and Psychomotor Outcomes: A Randomized Controlled Trial Study

**DOI:** 10.3390/dj12030063

**Published:** 2024-03-04

**Authors:** Valdy Hartono, Yoga Setiadharma, Aurelle Khadeeja Rizany, Benso Sulijaya, Robert Lessang, Natalina Haerani, Ette S. Tadjoedin, Sri Lelyati C. Masulili, Fatimah Maria Tadjoedin, Yuniarti Soeroso, Marie Rossini Carmela T. Lachica

**Affiliations:** 1 Periodontology Specialist Program, Department of Periodontology, Faculty of Dentistry, Universitas Indonesia, Jakarta 10430, Indonesia; valdy.hartono@ui.ac.id (V.H.); yoga.setiadharma@ui.ac.id (Y.S.); 2 Undergraduate Program, Faculty of Dentistry, Universitas Indonesia, Jakarta 10430, Indonesia; aurelle.khadeeja@ui.ac.id; 3 Department of Periodontology, Faculty of Dentistry, Universitas Indonesia, Jakarta 10430, Indonesia; robert_l@ui.ac.id (R.L.); natalina_perio@ui.ac.id (N.H.); ette.soraya@ui.ac.id (E.S.T.); lelyati@ui.ac.id (S.L.C.M.); fatimah.tadjoedin@ui.ac.id (F.M.T.); yuniarti@ui.ac.id (Y.S.); 4 College of Dentistry, University of the Philippines Manila, Manila 1000, Philippines; mtlachica@up.edu.ph

**Keywords:** mobile applications, periodontitis, periodontal index, dentistry, periodontology

## Abstract

This study aims to evaluate the clinical, cognitive, and psychomotor changes that emerged among patients with gingivitis and patients with periodontitis via mobile application assistance. Forty subjects were randomly and evenly distributed into test and control groups and were administered a professional mechanical plaque removal (PMPR) procedure. The clinical parameters examined were bleeding on probing (BoP), probing pocket depth (PPD), and the oral hygiene index (OHI-S). The test group was administered a mobile application intervention, namely PerioUICare, which provided users with continuous education and motivation and served as a daily dental hygiene reminder. The comparative results of the mean values of all parameters between the groups (inter-group) and within the groups (intra-group) after one- and three-month evaluations were analyzed. A one-month inter-group evaluation uncovered significant differences in the BoP, PPD, cognitive, and psychomotor scores of subjects with gingivitis and the BoP, OHI-S, cognitive, and psychomotor scores of subjects with periodontitis. A three-month inter-group evaluation revealed significant differences across all parameters except for PPD in subjects with periodontitis. The results of the intra-group comparison demonstrated statistically significant differences in all parameters in the test group but no significant differences in the cognitive and psychomotor scores of the control group. This study revealed that mobile application-based support for periodontal treatment can be considered effective.

## 1. Introduction

Periodontal disease, which encompasses gingivitis and periodontitis, is a group of diseases that involve plaque dysbiosis and host immunological responses resulting in inflammation and the destruction of the attachment apparatus [1]. The Global Burden of Disease Study stated that periodontitis, which was listed as the sixth most common disease in the world, has an average prevalence of 11.2%, with around 743 million people affected; the study also indicated that the need for treatment for the disease continues to increase [2]. A change to the classification of periodontal disease by the American Academy of Periodontology (AAP) and the European Federations of Periodontology (EFP) was recently introduced based on the severity and complexity of the disease (staging) and the risk of progression (grading) [1]. The clinical symptoms of periodontal disease may include redness, swelling, and bleeding at the gingivitis stage; if left untreated, the destruction will further escalate and cause attachment loss and pocket formation [3,4]. Hence, bleeding on probing (BoP) and probing pocket depth (PPD) have been established as important clinical parameters in detecting periodontal inflammation, while the absence or presence of clinical attachment loss (CAL) has been used to produce definitive diagnoses of gingivitis or periodontitis [1,3,4]. On the other hand, a relationship between oral hygiene, which was measured with the oral hygiene index simplified (OHI-S), and periodontitis has been found; specifically, patients with moderate or poor OHI-S have a significantly increased risk of experiencing periodontitis of up to two to five times [5].

The management of periodontal disease requires an understanding of the etiology, risk factors, and pathogenesis of the disease and the stages of periodontal treatment [6]. The first stage of periodontal treatment focuses on altering patient behavior by motivating patients to successfully eliminate supragingival biofilm and by controlling disease risk factors through dental health education (DHE), which necessitates adequate knowledge (a cognitive element) and the ability to maintain oral hygiene (a psychomotor element) [7]. According to several studies, these factors can be evaluated in three-month intervention periods [8,9]. Therefore, at this stage of treatment, the effectiveness of periodontal mobile application—used as an alternative means of educating, motivating, and reminding patients to clean their oral cavities repetitively and individually for one month and three months of use—was examined. At this juncture, supragingival scaling, a form of professional mechanical plaque removal (PMPR), was performed [6,7]. Moreover, a study conducted by Scribante et al. demonstrated that photodynamic therapy (PDT), photobiomodulation (PBM), and ozone as an adjunctive therapy may reduce the incidence of periodontal inflammation; this is known as a minimally invasive strategy [10,11]. The concept of minimally invasive periodontal therapy was one of the encouraging factors used in this study.

It is undeniable that the smartphone, a type of cell phone that is integrated with computer technology and other features such as an operating system, web browsing, and the ability to run mobile applications, has gained worldwide adoption and become the predominant communication mechanism [12,13]. The commercial, social, educational, and particularly health sectors have all witnessed significant changes due to smartphone technology [12]. The widespread use of smartphones across all age groups has underscored the degree to which this technology has exerted an impact on behavior change and is crucial in elevating patients’ quality of life [13,14]. Aside from various advantages, such as the efficiency and convenience of its mobility, a smartphone with health-related mobile applications allows patients to be monitored by health service providers in a manner that is more accessible and user-friendly and that reduces patients’ number of visits [15,16].

Mobile applications in the medical field have been widely published and used by the public [12,13,14,15,16,17,18]. Tobias et al. used “dental selfies” on smartphones to identify gingivitis cases using the modified gingival index (MGI) [16]. A randomized controlled trial by Alkadhi et al. demonstrated oral hygiene improvement in patients with fixed orthodontics using mobile applications [17]. Ng et al. have also proven the effect of educational web applications on oral and denture hygiene [18]. However, only a few studies have discussed the effectiveness of using mobile applications for patients with periodontal disease. Therefore, this randomized controlled trial study was conducted to determine the effectiveness of mobile application-based periodontal treatment by evaluating changes in the clinical parameters (BoP, PPD, and OHI-S) and the cognitive and psychomotor scores of patients with gingivitis and periodontitis.

## 2. Materials and Methods

### 2.1. Study Design

The present study was conducted as a randomized, double-blinded, controlled clinical trial. Forty patients with periodontal disease were randomly and evenly distributed into test and control groups at the Periodontics Clinic, Dental Teaching Hospital, Faculty of Dentistry, Universitas Indonesia, between April 2021 and December 2022. This study received written approval from the Dental Research Ethics Committee, Faculty of Dentistry, Universitas Indonesia, with letter number 01/Ethical Approval/FKGUI/I/2022 and Protocol Number 091241221. An explanation was given to all research subjects regarding the objectives of the research, the research flow, mobile application interventions, the expected advantages and disadvantages of participating, and guarantees of the confidentiality of subjects’ data. All study procedures proceeded after the subjects understood, were willing to participate, and had signed the consent letter. This trial was performed in accordance with the updated Declaration of Helsinki and conducted in agreement with the CONSORT (Consolidated Standards of Reporting Trials) guidelines [19], clinical trial registration number: ISRCTN12409366 (https://doi.org/10.1186/ISRCTN12409366).

### 2.2. Study Population

The inclusion criteria were as follows: (1) subjects with gingivitis or periodontitis; (2) age between 35 and 55 years; (3) no previous receipt of periodontal therapy during the previous six months; (4) Android system-based smartphone users. The exclusion criteria were as follows: (1) systemic conditions related to periodontal disease, such as cardiovascular disease, oral cancer, respiratory tract infection, and type 2 diabetes; (2) pregnancy or breastfeeding; (3) taking medication from a health provider; (4) current smokers [20]. The characteristics of the samples are presented in Table 1.

**Figure 1:**
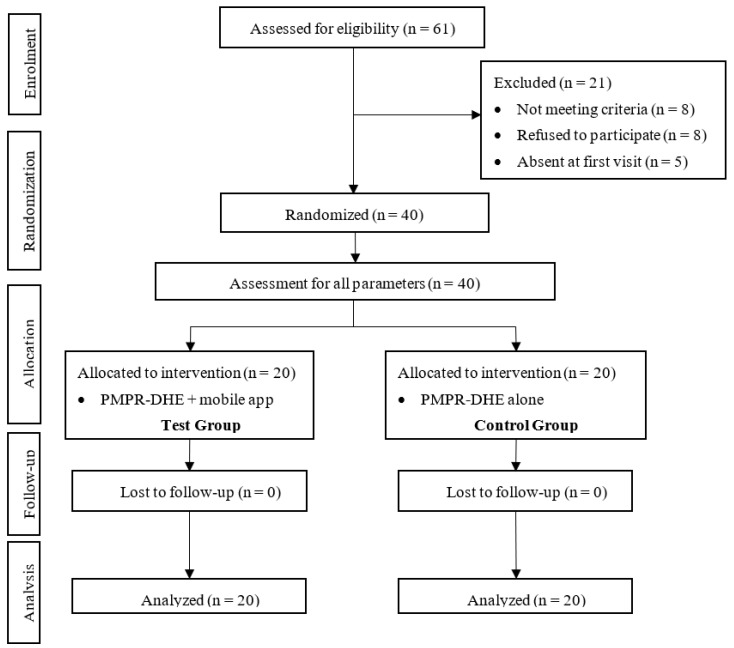
Flow diagram of the RCT study according to the CONSORT guidelines.

A total of 61 subjects were assessed for eligibility. Twenty-one subjects were excluded due to not fulfilling the criteria (n = 8), refusing to participate (n = 8), or being absent from the first visit (n = 5). After exclusion, 40 subjects were randomly distributed into the test group (n = 20) and the control group (n = 20), as indicated in Figure 1.

### 2.3. Clinical Examinations

Examinations of the clinical parameters and the cognitive and psychomotor scores were performed by two calibrated and masked investigators who were trained periodontology residents. Baseline clinical data, which included BoP, PPD, and OHI-S data, were recorded for all groups utilizing a periodontal probe (UNC-15, Hu-Friedy, Chicago, IL, USA). PPD was measured on six tooth surfaces, namely the distobuccal, mid-buccal, mesiobuccal, distolingual, mid-lingual, and mesiolingual surfaces of all teeth except for the third molars, and the average probing depth value was measured [21,22]. By assessing the presence of bleeding lasting up to 30 s, the BoP was recorded during the PPD measurement. Furthermore, a disclosing agent (GC Tri Plaque ID Gel, GC Corporation, Tokyo, Japan) was used to stain plaque and calculus on all tooth surfaces to be evaluated for OHI-S assessment [23]. The periodontal status of the subjects was determined to be gingivitis (BoP ≥ 10%; PPD ≤ 3 mm; no teeth with interdental CAL) or periodontitis (BoP ≥ 10%; PPD > 3 mm; interdental CAL with a minimum of two non-adjacent teeth) [24].

Using BoP and PPD as the reference values, the inter- and intra-rater reliability was assessed using the analysis of the intraclass correlation coefficient (ICC). The intra-rater reliability indicated a very strong level of agreement for both examiners (BoP: 0.998 for the first examiner and 0.999 for the second examiner; PPD: 1 for both the first and second examiners). The inter-rater reliability also demonstrated a very strong level of agreement (BoP: 0.995; PPD: 1).

Cognitive scores were recorded using pre-test questions at the baseline and post-test questions during the evaluation visits, while psychomotor scores were assessed using a checklist of oral hygiene procedures (Table S1). The cognitive measuring instrument (pre-test and post-test questions) is available in the supplementary data. The two measuring instruments were checked for validation.

The evaluation of clinical parameters and cognitive and psychomotor scores was performed at the baseline and after one and three months of using the mobile application. The collected data were entered into the periodontal mobile application via a back-end website by a data entry operator.

### 2.4. Sample Size Calculation

The sample size was calculated using G* Power statistical software (version 3.1.9.7, Heinrich Heine, Universität Düsseldorf, Germany) [25], assuming an effect size of 0.8 with α = 0.05 and a power level of 0.8 for two independent study groups, and a continuous primary endpoint was calculated. It was determined that each group would require a minimum of 12 subjects. Concerning the variable probing pocket depth (the primary outcome), the expected difference between the means was supposed to be 0.1305^10^ and potential dropouts 20%; therefore, 20 patients per group could be enrolled in this study. In the present study, there were two groups, the test (PMPR-DHE + mobile application) and control (PMPR-DHE alone) groups. By means, at least 40 subjects were involved in the study at three time points (the baseline, one month, and three months). The calculation was performed with a Sample Size Calculator ClinCalc (ClinCalc LLC., Chicago, IL, USA).

### 2.5. Randomization

Forty subjects were listed and evenly distributed between the test (PMPR-DHE + mobile application) and control (PMPR-DHE alone) groups by a masked researcher who was not involved in the clinical examination. This was done using a systematic random sampling method, which distributed subjects with odd numbers to the test group and those with even numbers to the control group [10]. The subjects were concealed from both the treating clinician and the data entry operator, thus precluding bias in evaluating the collected data.

### 2.6. Treatment and Interventions

PMPR and DHE were performed for all subjects after the clinical parameters, and the cognitive and psychomotor scores were recorded by the calibrated and masked investigator, who was a trained periodontology resident. A mobile periodontal application intervention was only implemented for subjects who belonged to the test group. First, the subjects were instructed to download and install a mobile app (PerioUICare, Universitas Indonesia, Jakarta, Indonesia); the subjects were then directed to input a username and password. For every visit, clinical periodontal parameters, which were recorded, were displayed inside the app to ensure that the subjects were aware of whether their periodontal condition had improved or deteriorated. Additionally, the app served as a daily dental hygiene reminder for users, as well as a constant source of education and motivation for periodontal health maintenance. Instructions on how to perform the proper way of brushing and the use of complimentary dental hygiene tools, notably interdental brushes and dental floss, were given simultaneously with the daily reminder sent for users through the app twice a day (every morning and every night). Moreover, dental health education was provided to users three times a week in the form of interactive posters.

### 2.7. Statistical Analysis

**Figure 2:**
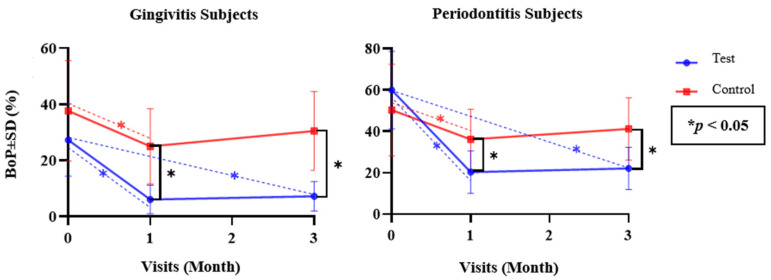
Mean BoP Value Differences in Both Groups over Time.

**Figure 3:**
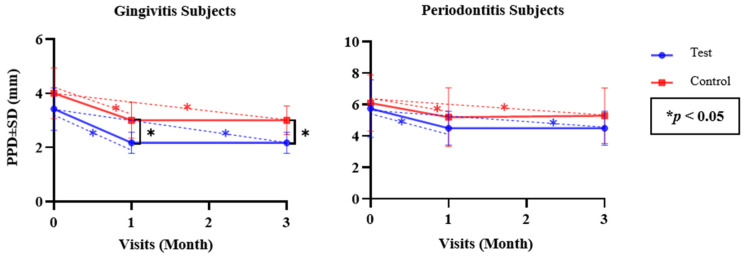
Mean PPD Value Differences in Both Groups over Time.

**Figure 4:**
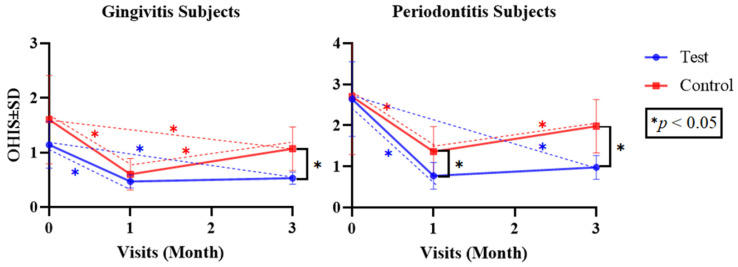
Mean OHI-S Value Differences in Both Groups over Time.

**Figure 5:**
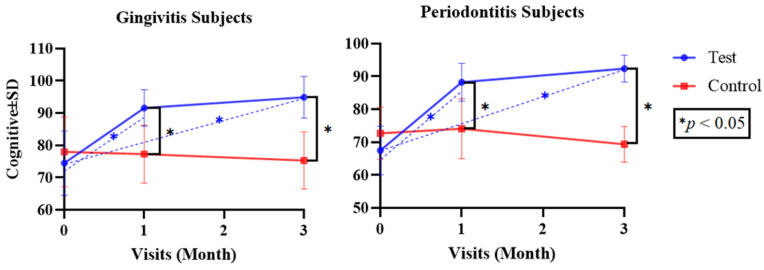
Mean Cognitive Score Differences in Both Groups over Time.

**Figure 6:**
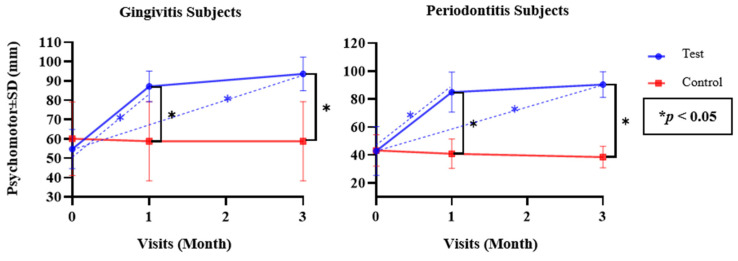
Mean Psychomotor Score Differences in Both Groups over Time.

Since the numerical parameters exhibited both normal and non-normal data distribution, as confirmed with the Shapiro–Wilk test, both parametric and non-parametric approaches were applied. Independent *t*-tests and Mann–Whitney U tests were used to analyze differences between the test and control groups (inter-group), while repeated measures using ANOVA and Friedman tests were used to analyze differences in each test and control group (intra-group) for the parametric and non-parametric tests, respectively (Table 2 and Table 3). Differences between each follow-up visit were determined via a post hoc Bonferroni test (Figure 2, Figure 3, Figure 4, Figure 5 and Figure 6). The statistical software program SPSS 26.0 for Windows (IBM, Chicago, IL, USA) was used to execute all statistical analyses, with *p* < 0.05 being considered statistically significant. The graphs were made using the Prism 9.0.0 software (GraphPad, Boston, MA, USA).

## 3. Results

Comparisons of all parameters across all timelines are presented in Table 2, Table 3, Table 4 and Table 5. All patients successfully completed the study in an uneventful manner and without any adverse events.

No significant differences were observed between groups at the baseline for all the parameters. A one-month evaluation uncovered significant differences in the BoP (*p* = 0.001), PPD (*p* = 0.007), cognitive (*p* = 0.000), and psychomotor scores (*p* = 0.001) of subjects with gingivitis and in the BoP (*p* = 0.02), OHI-S (*p* = 0.043), cognitive (*p* = 0.003), and psychomotor scores (*p* = 0.000) of subjects with periodontitis. A three-month evaluation revealed significant differences for all parameters (*p* < 0.05) except for PPD (*p* = 0.315) in subjects with periodontitis.

Over time, both the test and control groups of subjects with gingivitis and periodontitis exhibited significant improvements in the clinical parameters (*p* < 0.05). However, regarding the cognitive and psychomotor scores, there were no significant differences in the control group for either patients with gingivitis (*p* = 0.227 and *p* = 1 for cognitive and psychomotor scores) or patients with periodontitis (*p* = 0.095 and *p* = 0.174 for cognitive and psychomotor scores); only the test group exhibited significant differences for either patients with gingivitis (*p* = 0.000 and *p* = 0.000 for cognitive and psychomotor scores) or patients with periodontitis (*p* = 0.001 and *p* = 0.000 for cognitive and psychomotor scores, respectively).

Post hoc tests were utilized to determine the differences between each follow-up visit (Figure 2, Figure 3, Figure 4, Figure 5 and Figure 6). Figure 2 illustrates the differences in the mean BoP values of subjects with gingivitis and subjects with periodontitis in the test and control groups at the baseline and at the one- and three-month follow-up visits. A post hoc analysis of the test group revealed a significant reduction in the mean BoP values between the baseline and the one-month evaluation (*p* = 0.000 and *p* = 0.000 for gingivitis and periodontitis), representing a non-significant increase from the one-month evaluation to the three-month evaluation (*p* = 0.227 and *p* = 0.119 for gingivitis and periodontitis) and a significant reduction between the baseline and three-month evaluation (*p* = 0.001 and *p* = 0.001 for gingivitis and periodontitis). On the other hand, the control group exhibited a significant reduction in mean BoP values only between the baseline and the one-month evaluation (*p* = 0.001 and *p* = 0.039 for gingivitis and periodontitis, respectively) (Figure 2).

Figure 3 indicates the differences in the mean PPD values of subjects with gingivitis and periodontitis in the test and control groups at the baseline and at the one- and three-month follow-up visits. A post hoc analysis of the test group revealed a significant reduction in the mean PPD values between the baseline and the one-month evaluation (*p* = 0.007 and *p* = 0.024 for gingivitis and periodontitis), a stable PPD value from the one-month evaluation to the three-month evaluation (*p* = 1 and *p* = 1 for gingivitis and periodontitis), and a significant reduction between the baseline and the three-month evaluation (*p* = 0.007 and *p* = 0.024 for gingivitis and periodontitis). The same applied to the control group, except that there was a non-significant increase in the mean PPD value from the one-month evaluation to the three-month evaluation (*p* = 0.943 and *p* = 1; for gingivitis and periodontitis, respectively) (Figure 3).

Figure 4’s OHI-S scores describe the differences in the mean OHI-S values for subjects with gingivitis and periodontitis in the test and control groups at the baseline and at the one- and three-month follow-up visits. The post hoc analysis of the test group revealed a significant reduction in the mean OHI-S values between the baseline and the one-month evaluation (*p* = 0.000 and *p* = 0.001 for gingivitis and periodontitis), a non-significant increase in value from the one-month evaluation to the three-month evaluation (*p* = 0.922 and *p* = 0.401 for gingivitis and periodontitis), and a significant reduction between the baseline and the three-month evaluation (*p* = 0.013 and *p* = 0.037 for gingivitis and periodontitis). Patients with gingivitis in the control group exhibited significant reductions in their mean OHI-S values from the baseline to the one-month and three-month evaluations (*p* = 0.000); however, there was also a significant increase in OHI-S values from the one-month evaluation to the three-month evaluation (*p* = 0.044). The same applied to patients with periodontitis, except that there was no significant difference between the baseline and three-month evaluations (*p* = 0.259) (Figure 4).

Figure 5 indicates the differences between the mean cognitive scores for subjects with gingivitis and subjects with periodontitis in the test and control groups at the baseline and at the one- and three-month follow-up visits. A post hoc analysis of the test group revealed a significant increase in mean cognitive scores from the baseline to the one-month evaluation (*p* = 0.007 and *p* = 0.037 for gingivitis and periodontitis), a non-significant increase in value from the one-month evaluation to the three-month evaluation (*p* = 0.662 and *p* = 0.952 for gingivitis and periodontitis), and a significant increase between the baseline and the three-month evaluation (*p* = 0.000 and *p* = 0.001 for gingivitis and periodontitis), while there were no significant differences in the control group over time (*p* > 0.05).

Figure 6 illustrates the differences in the mean psychomotor scores for subjects with gingivitis and subjects with periodontitis in the test and control groups at the baseline and at the one- and three-month follow-up visits. The post hoc analysis of the test group revealed a significant increase in the mean psychomotor scores from the baseline to the one-month evaluation (*p* = 0.009 and *p* = 0.001 for gingivitis and periodontitis), a non-significantly increased value from the one-month evaluation to the three-month evaluation (*p* = 0.459 and *p* = 0.099; for gingivitis and periodontitis), and a significant increase between the baseline and the three-month evaluation (*p* = 0.000 and *p* = 0.000 for gingivitis and periodontitis); conversely, there were no significant differences in the control group over time (*p* > 0.05).

## 4. Discussion

The treatment for periodontal disease focused on removing the etiological factors in the form of PMPR, as well as encouraging patient motivation [26]. The key to achieving periodontal tissue health is to organize an individual and repetitive approach, namely by providing oral hygiene instructions tailored to patients’ needs and motivating them on a regular basis to increase their knowledge about the disease and to understand the benefits resulting from their behavior changes [27]. Therefore, this study was conducted in accordance with this understanding, which underscored that patients’ cognitive and psychomotor characteristics can be improved through personalized and repeated motivations and instructions by means of periodontal mobile applications, which might exert a positive impact on periodontal tissue without the need for routine in-office visits.

Table 1 indicates that 40 total subjects were included; these subjects were divided randomly with 20 subjects each in the test and control groups. The mean age of the subjects was 46.45 ± 6.863 years in the test group and 43.90 ± 7.532 years in the control group. The incidence of periodontal disease is consistent with increasing age. However, at an advanced age, elderly people experience limitations in being able to keep up with technological advances [13,14]. Therefore, we restricted the age range to 35 to 55 years to obtain desirable and homogeneous samples. The test groups with senior high school and bachelor’s education levels totaled 14 subjects and 6 subjects, respectively; conversely, the control groups had four subjects and 16 subjects, respectively.

Based on an examination of the periodontal status at the baseline, there were twelve patients with gingivitis and eight patients with periodontitis in the test group; meanwhile, a balanced number was obtained, specifically ten for both patients with gingivitis and patients with periodontitis in the control group. We did not classify subjects with periodontitis into certain stages and grades, as suggested by the AAP/EFP at the 2017 World Workshop. We believed that patients with stages I–IV and grades A–C of periodontitis must undergo initial periodontal therapy or the first step of the EFP S3-level clinical practice guideline, which includes PMPR and oral hygiene motivations and instructions [7]. We aimed to uncover the effectiveness of mobile application-based periodontal therapy in general. Therefore, in this study, we focused on subjects with gingivitis and subjects with periodontitis.

The periodontal mobile application used in this study is only registered on the Google Play Store, which can only be downloaded and used on Android-operated smartphones. Hence, only subjects who had Android-based smartphones were included in this study. The inability of the mobile app to compel subjects to use the full functionality of the app was another limitation of this study; thus, the clinical parameters and the cognitive and psychomotor scores were the objective standards to determine whether any of the test group subjects genuinely engaged in the study.

The results of the inter-group comparison at the baseline and the mean values of BoP, PPD, and OHI-S in the test and control groups with gingivitis were 27.27 ± 12.94, 3.42 ± 0.79, and 1.14 ± 0.43 versus 37.62 ± 17.98, 4 ± 0.94, and 1.60 ± 0.81, respectively, while the results for the test and control groups with periodontitis were 59.92 ± 18.77, 5.75 ± 1.83, and 2.64 ± 0.91 versus 50.24 ± 22.15, 6.1 ± 1.79, and 2.7 ± 1.41, respectively. These results indicated that there were no significant differences in the mean values of all parameters between the subjects in the test and control groups (*p* > 0.05). 

Following one month of use, only subjects with gingivitis were found to have a lower mean PPD value in the test group compared to the control group, while test group subjects with gingivitis and periodontitis both had lower mean BoP values that were statistically significant compared to the control group (*p* = 0.001 and *p* = 0.020 for gingivitis and periodontitis at the one-month evaluation). The three-month evaluation revealed consistent BoP outcomes (*p* = 0.000 and *p* = 0.008 for gingivitis and periodontitis at the three-month evaluation). The difference in the PPD values, which was not significant between the test and control groups for patients with periodontitis, was in accordance with the conditions described by Gul et al. (2022), namely that periodontitis caused the destruction of the periodontal tissue, which was characterized by the presence of periodontal pockets and the loss of attachment; it exhibited an irreversible condition, as opposed to gingivitis, which allows the periodontal tissue to heal and return to its original state after initial therapy [28]. The treatment of residual pockets requires a third stage of periodontal therapy, namely surgical intervention, such as open flap debridement, which can be followed by regenerative or resective periodontal therapy, according to the type of alveolar bony defect [29,30].

The inter-group comparison results revealed that there was no significant difference in the mean values for OHI-S between the test and control groups for subjects with gingivitis after one month of use (*p* = 0.283); however, there was a significant difference in the mean values of OHI-S between the test and control groups for subjects with gingivitis after three months of use *(p* = 0.002). Following the one- and three-month interventions, there was a significant difference in the mean values of OHI-S between the test and control groups for subjects with periodontitis (*p* = 0.043 and *p* = 0.001 for the one- and three-month evaluations). Similar outcomes regarding the value of the oral hygiene index were also demonstrated by Marchetti et al. (2018), who proved that the use of mobile applications in providing periodic repetitions of motivation and education is an effective strategy [31]. A recent study revealed that mobile applications can be utilized as an additional method for treating periodontal disease, a source of knowledge regarding dental and oral health, and a means of enhancing dental and oral hygiene [32]. Poor oral hygiene mostly occurs as a result of inadequate knowledge and skills when performing oral hygiene. When oral hygiene is maintained correctly in accordance with the knowledge that has been provided, when complemented by professional maintenance care, effective periodontal treatment can be achieved [33].

The intra-group comparison results after a one-month intervention indicated that the mean BoP and PPD values for patients with gingivitis in the test and control groups underwent a statistically significant reduction (*p* < 0.05) to 6.04 ± 5.08 and 2.17 ± 0.39 versus 24.99 ± 13.44 and 3 ± 0.67, respectively. For patients with periodontitis, the mean BoP and PPD values in the test and control groups also decreased statistically significantly (*p* < 0.05) to 20.30 ± 10.25 and 4.5 ± 1.07 versus 36.07 ± 14.56 and 5.2 ± 1.87, respectively. This outcome was explained by Jentsch et al. (2019) in their study, which indicated that supra- and subgingival instrumentation alone proved to exert a significant positive impact on BoP and PPD [34]. This was demonstrated by the study’s findings, which revealed that, despite the absence of any mobile app intervention, the mean BoP and PPD values of patients with gingivitis and periodontitis both exhibited considerable reductions. However, a greater decrease was found in the test group. The greater decrease in the mean BoP and PPD values in the test group was supported by a study conducted by Milosavljevic et al. (2021), which stated that providing education and motivation in a manner that allows patients to maintain oral hygiene routines is pivotal to the success of therapy [29].

At the three-month follow-up visit, the results indicated that the mean BoP value for the test and control groups underwent a non-significant increase (*p* > 0.05) to 7.22 ± 5.26 and 30.48 ± 14.05 for patients with gingivitis and 22.04 ± 10.27 and 41.18 ± 15.10 for patients with periodontitis. On the other hand, the mean PPD value in the test group remained stable at 2.17 ± 0.39 for patients with gingivitis and 4.5 ± 1.07 for patients with periodontitis. However, there was a slight increase (*p* > 0.05) in the control group to 3.5 ± 0.53 and 5.3 ± 1.77, respectively, for patients with gingivitis and patients with periodontitis. Although not significant, the increase in the value of the clinical inflammation parameters mentioned above can be explained by a study published by Lang et al. (2018) that stated that chronic inflammation will always occur in the periodontal tissue; in other words, it is highly uncommon to have pristine periodontal health, which is the absence of redness, edema, and BoP [35].

One of the interesting results of this study was the stable PPD value from the one- to three-month evaluation visit for both patients with gingivitis and patients with periodontitis in the test group. This was in accordance with a study conducted by Arweiler et al. (2018), which stated that, in a pocket less than or equal to 5 mm, if the patient possesses adequate knowledge and the psychomotor ability to perform oral hygiene, it is possible to achieve a stable condition, and they may not need additional therapy [36]. However, reaching the stage where the patient can clean the oral cavity optimally necessitates proper education and motivation by professionals, as well as dexterity and compliance from the patient [37].

The classification of periodontal health and disease at the 2017 World Workshop stated that a BoP value below 10% indicates clinical health gingiva [38]. The mean BoP value of patients with gingivitis in the test group was found to decrease below 10% after one and three months of the intervention. This was consistent with a study conducted by Murakami et al. (2017), which explained the reversibility of gingivitis to be clinically healthy if the strategy for eliminating biofilm is executed correctly [39]. This means that mobile application-based periodontal treatment as an additional educational and motivational method is the appropriate treatment strategy; as observed in the control group, there was no reduction in the mean BoP value to below 10%, and the subjects were still experiencing gingivitis. Moreover, according to Stein et al. (2018), delivering oral health education and motivation on a regular basis, similar to mobile apps, is effective in lowering plaque levels, which are the primary cause of periodontal disease [40].

The intra-group comparison results demonstrated that there was a significant difference in the mean values of OHI-S in both the test and control groups for subjects with gingivitis, as well as subjects with periodontitis, after one and three months of the intervention (*p* < 0.05). A thorough understanding of routine care and strict adherence to recall schedules are highly important, and this aspect is related to patients’ knowledge regarding the success of long-term periodontal stability [41]. The use of mobile applications, when compared to conventional methods such as brochures, will yield more benefits in terms of their usefulness in disseminating knowledge about oral health information due to the scheduled reminder function in the form of notifications that can be received anytime and anywhere [42]. Reminders and education in the mobile applications that were provided repeatedly have succeeded in motivating patients to improve their oral hygiene, increased the success of treatment, and reduced treatment visits [18].

Regarding the results of the inter-group comparison at the baseline, the mean values for the cognitive element in the test and control groups with gingivitis were 74.5 ± 9.94 and 78 ± 10.8, respectively, while the values for the test and control groups with periodontitis were 67.5 ± 7.43 and 72.7 ± 7.96, respectively. There was no significant difference in the cognitive scores between the test group and the control group for either subjects with gingivitis or subjects with periodontitis at the initial examination (*p* > 0.05).

The inter-group comparison results revealed that there was a significant difference in the mean values of cognitive aspects between the test and control groups for both subjects with gingivitis and subjects with periodontitis after one and three months of the intervention (*p* = 0.000 and *p* = 0.003 for gingivitis and periodontitis at the one-month evaluation; *p* = 0.000 and *p* = 0.000 for gingivitis and periodontitis at the three-month evaluation). The patients’ periodontal and cognitive statuses were correlated because patients can achieve a stable periodontal condition by increasing their awareness regarding maintaining oral hygiene, and professionals must be able to communicate effectively so that instructions are delivered optimally [41,43]. A study by Stein et al. (2018) demonstrated that repeated motivation and knowledge concerning oral dental health can effectively reduce the amount of plaque (an indicator of the oral hygiene index), which is the main cause of periodontal disease [40].

The intra-group comparison results for subjects with gingivitis and subjects with periodontitis after one and three months of the intervention revealed that there was a significant difference in the mean values of cognitive aspects for the test group (*p* < 0.05), but there was no significant difference in the mean values of cognitive aspects in the control group (*p* > 0.05). Patients with periodontal disease require a thorough understanding of the condition of their periodontal tissue, particularly in relation to maintaining proper dental hygiene and adhering to scheduled visit intervals [41]. Patient knowledge (cognitive aspects) exerts an important impact on maintaining proper dental and oral hygiene and the effectiveness of plaque control [44].

A baseline comparison demonstrated that mean psychomotor scores in the test and control groups with gingivitis were 54.75 ± 10.18 and 60.1 ± 19.05, respectively, while the scores for the test and control groups with periodontitis were 42.88 ± 17.47 and 43.2 ± 11.31, respectively. These results indicated that there were no significant differences in the psychomotor scores between subjects in the test and control groups at the baseline (*p* > 0.05).

A one-month evaluation revealed that the mean psychomotor scores in the test group with gingivitis and periodontitis had increased significantly, while the mean scores declined, albeit not significantly, in the control group. At this point, the test group exhibited a significantly higher mean psychomotor score than the control group (*p* = 0.001 and *p* = 0.000 for gingivitis and periodontitis, respectively). The three-month evaluation showed that the mean psychomotor scores in the test group with gingivitis and periodontitis continued to increase, although not significantly, while the control group with gingivitis did not experience a change, and subjects with periodontitis underwent a non-significant reduction. At this point, the test group exhibited a significantly higher mean psychomotor score than the control group (*p* = 0.000 and *p* = 0.000 for gingivitis and periodontitis, respectively). According to the aforementioned findings, the mean psychomotor scores for the patients with gingivitis and the patients with periodontitis in the control group decreased slightly but not considerably after the one- and three-month evaluations. This was in accordance with the results of Petrauskiene et al. (2019), who found that patients typically exhibit low motivation and poor long-term adherence in relation to maintaining proper oral hygiene [45]. Therefore, the periodontal mobile application can be a solution to increasing patient motivation through individual and repetitive reminders, encouragement, and educational features.

The results of this study were consistent with the randomized controlled trial conducted by Marchetti et al. (2018), which explained that providing periodic motivational and educational repetition using this mobile application as a medium is an effective strategy to ensure that the benefits of the content are adequately fixed, as is evident in the significant difference between the test group and the control group for the oral hygiene index [32]. Furthermore, in their study, Geisinger et al. (2019) stated that instructions for recommending oral hygiene techniques should vary or be individualized based on the clinical presentation of the oral cavity and the patient’s risk assessment [46]. In line with these studies, the periodontal mobile application that was used as a means of reminders, motivation, and education that is individual and repetitive was able to increase statistically significant psychomotor scores for patients with gingivitis and periodontitis after one and three months of use; it was proven that the test group exhibited a significantly higher mean psychomotor score than the control group.

A study that examined the effect of a reinforced oral hygiene instruction program on the gingiva health of patients using fixed orthodontic appliances, conducted by Singla et al. (2019), demonstrated that there was an improvement in the subjects’ psychomotor ability to clean the oral cavity, as indicated by a significant decrease in the mean plaque score (PI) and gingival index (GI) in the test group after four and eight weeks of using the program [47]. In addition, Williams et al. (2018) conducted a study that compared the effectiveness of oral hygiene instructions provided through verbal instructions and the use of computer assistance, in which a lower plaque score was obtained in the group that was given the intervention of computer-assisted oral cleaning instructions, although it was only significant in subjects aged 50 years and under, compared to the group that was only offered verbal instructions by professionals [48].

It can be concluded that permanent improvements in psychomotor scores cannot be achieved merely by providing motivation and education that is not individual and repetitive in nature. A periodic motivational and educational approach, known as periodic reinforcement, is necessary [39,41]; this involved the use of a periodontal mobile application in the present study. Additionally, some recently introduced compounds have been demonstrated to have a significant influence on the oral environment [49]. This was consistent with the results of the current study indicating that the mean psychomotor score in the test group with both subjects with gingivitis and subjects with periodontitis increased significantly, whereas there were no significant differences in the mean psychomotor scores in the control group among both subjects with gingivitis and subjects with periodontitis after the one- and three-month evaluations. The use of lysates and postbiotics can modify clinical and microbiological parameters in periodontal patients; these products should be considered in future trials in combination with the use of mobile applications [50,51]. According to the present findings, periodontal treatment delivered via a mobile application was successful in promoting psychomotor scores.

The limitation of our study was the range of the participants’ age, which was limited to young and early adulthood. The utility of a mobile application that is more applicable to a segmented group of people and technologically friendly is undeniable. Also, the educational background of the enrolled subjects in this study was homogeneous. It may be assumed that people with a higher level of education may have a higher chance of adapting to a new concept than those with a lower one.

## 5. Conclusions

This study demonstrated that mobile application-based periodontal treatment, as a component of the first stage of periodontal therapy, can be considered effective in improving clinical, cognitive, and psychomotor factors. This mobile app may be considered or offered as an additional periodontal treatment alternative. Collaboration between stakeholders, decision-makers, and healthcare providers is necessary to introduce this concept as a preventive and promotive approach to reducing periodontal disease.

## Figures and Tables

**Table 1:** Age, education, and periodontal status distribution throughout the study sample.

	Group	
	Test (n = 20)	Control (n = 20)
Age ± SD (years)	46.45 ± 6.863	43.90 ± 7.532
Education level		
Senior high school	14	12
Bachelor’s	6	8
Periodontal status		
Gingivitis	12	10
Periodontitis	8	10

**Table 2:** Inter-group Comparison Outcomes at Baseline and for Each Follow-up Visit among Patients with Gingivitis.

	Group	Baseline	*p*-Value	1 Month	*p*-Value	3 Months	*p*-Value
BoP ± SD (%)	Test (n = 20)	27.27 ± 12.94	0.180 ^δ^	6.04 ± 5.08	0.001 ^†^ *	7.22 ± 5.26	0.000 ^†^ *
	Control (n = 20)	37.62 ± 17.98		24.99 ± 13.44		30.48 ± 14.05	
PPD ± SD (mm)	Test (n = 20)	3.42 ± 0.79	0.203 ^δ^	2.17 ± 0.39	0.007 ^δ^ *	2.17 ± 0.39	0.000 ^δ^ *
	Control (n = 20)	4 ± 0.94		3 ± 0.67		3.5 ± 0.53	
OHI-S ± SD	Test (n = 20)	1.14 ± 0.43	0.127 ^†^	0.47 ± 0.12	0.283 ^δ^	0.53 ± 0.11	0.002 ^†^ *
	Control (n = 20)	1.60 ± 0.81		0.6 ± 0.29		1.07 ± 0.4	
Cognitive ± SD	Test (n = 20)	74.5 ± 9.94	0.438 ^†^	91.58 ± 5.65	0.000 ^†^ *	94.92 ± 6.43	0.000 ^†^ *
	Control (n = 20)	78 ± 10.8		77.3 ± 8.99		75.3 ± 8.82	
Psychomotor ± SD	Test (n = 20)	54.75 ± 10.18	0.674 ^δ^	87.17 ± 7.9	0.001 ^δ^ *	93.58 ± 8.72	0.000 ^δ^ *
	Control (n = 20)	60.1 ± 19.05		58.8 ± 20.46		58.8 ± 20.46	

^†^ Independent *t*-test (inter-group parametric test); ^δ^ Mann–Whitney U test (inter-group non-parametric test); * *p*-value < 0.05, statistically significant.

**Table 3:** Intra-Group Comparison Outcomes at Baseline and for Each Follow-up Visit among Patients with Gingivitis.

	Group	Baseline	1 Month	3 Months	*p*-Value
BoP ± SD (%)	Test (n = 20)	27.27 ± 12.94	6.04 ± 5.08	7.22 ± 5.26	0.000 ^#^ *
	Control (n = 20)	37.62 ± 17.98	24.99 ± 13.44	30.48 ± 14.05	0.001 ^^^ *
PPD ± SD (mm)	Test (n = 20)	3.42 ± 0.79	2.17 ± 0.39	2.17 ± 0.39	0.000 ^^^ *
	Control (n = 20)	4 ± 0.94	3 ± 0.67	3.5 ± 0.53	0.005 ^^^ *
OHI-S ± SD	Test (n = 20)	1.14 ± 0.43	0.47 ± 0.12	0.53 ± 0.11	0.000 ^^^ *
	Control (n = 20)	1.60 ± 0.81	0.6 ± 0.29	1.07 ± 0.4	0.000 ^#^ *
Cognitive ± SD	Test (n = 20)	74.5 ± 9.94	91.58 ± 5.65	94.92 ± 6.43	0.000 ^#^ *
	Control (n = 20)	78 ± 10.8	77.3 ± 8.99	75.3 ± 8.82	0.227 ^#^
Psychomotor ± SD	Test (n = 20)	54.75 ± 10.18	87.17 ± 7.9	93.58 ± 8.72	0.000 ^^^ *
	Control (n = 20)	60.1 ± 19.05	58.8 ± 20.46	58.8 ± 20.46	1

^#^ repeated measures ANOVA (intra-group parametric test); ^^^ Friedman test (intra-group non-parametric test); * *p*-value < 0.05, statistically significant.

**Table 4:** Inter-Group Comparison Outcomes at Baseline and for Each Follow-up Visit among Patients with Periodontitis.

	Group	Baseline	*p*-Value	1 Month	*p*-Value	3 Months	*p*-Value
BoP ± SD (%)	Test (n = 20)	59.92 ± 18.77	0.340 ^†^	20.30 ± 10.25	0.020 ^†^ *	22.04 ± 10.27	0.008 ^†^ *
	Control (n = 20)	50.24 ± 22.15		36.07 ± 14.56		41.18 ± 15.10	
PPD ± SD (mm)	Test (n = 20)	5.75 ± 1.83	0.408 ^δ^	4.5 ± 1.07	0.460 ^δ^	4.5 ± 1.07	0.315 ^δ^
	Control (n = 20)	6.1 ± 1.79		5.2 ± 1.87		5.3 ± 1.77	
OHI-S ± SD	Test (n = 20)	2.64 ± 0.91	0.915 ^†^	0.77 ± 0.33	0.043 ^δ^ *	0.98 ± 0.29	0.001 ^†^ *
	Control (n = 20)	2.7 ± 1.41		1.36 ± 0.61		1.98 ± 0.65	
Cognitive ± SD	Test (n = 20)	67.5 ± 7.43	0.175 ^†^	88.25 ± 5.73	0.003 ^†^ *	92.38 ± 4.1	0.000 ^†^ *
	Control (n = 20)	72.7 ± 7.96		74.1 ± 9.16		69.4 ± 5.46	
Psychomotor ± SD	Test (n = 20)	42.88 ± 17.47	0.962 ^†^	84.88 ± 14.33	0.000 ^†^ *	90.38 ± 9.18	0.000 ^δ^ *
	Control (n = 20)	43.2 ± 11.31		40.8 ± 10.59		38.5 ± 7.78	

^†^ Independent *t*-test (inter-group parametric test); ^δ^ Mann–Whitney U test (inter-group non-parametric test); * *p*-value < 0.05, statistically significant.

**Table 5:** Intra-group Comparison Outcomes at Baseline and for Each Follow-up Visit among Patients with Periodontitis.

	Group	Baseline	1 Month	3 Months	*p*-Value
	Test (n = 20)	59.92 ± 18.77	20.30 ± 10.25	22.04 ± 10.27	0.000 ^#^ *
BoP ± SD (%)	Control (n = 20)	50.24 ± 22.15	36.07 ± 14.56	41.18 ± 15.10	0.024 ^#^ *
PPD ± SD (mm)	Test (n = 20)	5.75 ± 1.83	4.5 ± 1.07	4.5 ± 1.07	0.002 ^^^ *
	Control (n = 20)	6.1 ± 1.79	5.2 ± 1.87	5.3 ± 1.77	0.001 ^^^ *
	Test (n = 20)	2.64 ± 0.91	0.77 ± 0.33	0.98 ± 0.29	0.001 ^^^ *
OHI-S ± SD	Control (n = 20)	2.7 ± 1.41	1.36 ± 0.61	1.98 ± 0.65	0.01 ^#^ *
Cognitive ± SD	Test (n = 20)	67.5 ± 7.43	88.25 ± 5.73	92.38 ± 4.1	0.001 ^#^ *
	Control (n = 20)	72.7 ± 7.96	74.1 ± 9.16	69.4 ± 5.46	0.095 ^#^
Psychomotor ± SD	Test (n = 20)	42.88 ± 17.47	84.88 ± 14.33	90.38 ± 9.18	0.000 ^#^ *
	Control (n = 20)	43.2 ± 11.31	40.8 ± 10.59	38.5 ± 7.78	0.174 ^^^

^#^ repeated measures ANOVA (intra-group parametric test); ^^^ Friedman test (intra-group non-parametric test); * *p*-value < 0.05, statistically significant.

## Data Availability

The raw data supporting the conclusions of this article will be made available by the authors on request.

## Supplementary Materials

The following supporting information can be downloaded at: https://www.mdpi.com/article/10.3390/dj12030063/s1, Cognitive Measuring Instrument (pre-test and post-test questions) in Indonesia. Table S1: Psychomotor Measuring Instrument (checklist of oral hygiene procedures).
