# Supplementary material for: Mobile Application-Based Support for Periodontal Treatment Improves Clinical, Cognitive, and Psychomotor Outcomes: A Randomized Controlled Trial Study
Source: Dent J (Basel). 2024 Mar 4;12(3):63. doi: 10.3390/dj12030063 (PMC10969222; doi:10.3390/dj12030063)
Supplement: Supplementary file 1 [file dentistry-12-00063-s001.zip › dentistry-2798787-supplementary.pdf]

dentistry journal
MDPI

Cognitive Measuring Instrument (pre-test and post-test questions) in Indonesia (English)

1. Ciri – ciri gusi yang sehat adalah (Sign of a healthy gums) …
   a. Gusi terlihat membesar (Swelling)
   b. Gusi mengeluarkan nanah (Pus comes out)
   c. Gusi berwarna merah muda (Pink coral in color)

2. Yang merupakan gejala penyakit gusi adalah (Below are signs of gums disease) …
   a. Gusi mudah berdarah (Easy to bleed)
   b. Gusi bengkak (Swelling)
   c. Gusi mengerut/turun (Gum recession)
   d. Semua benar (All is correct)

3. Gusi dapat mengalami perubahan warna apabila sering (Gum discoloration might be due to) …
   a. Merokok (Smoking)
   b. Mengunyah sirih (Betel nut chewing)
   c. Minum kopi (Coffee)

4. Penyebab terjadinya gusi bengkak dan mudah berdarah adalah (The etiology of gingival swelling and bleeding)…
   a. Penumpukan plak pada gigi (Dental plaque accumulation)
   b. Pasta gigi yang salah (Toothpaste misuse)
   c. Makan permen (Candies)

5. Yang bukan termasuk salah satu faktor risiko di dalam rongga mulut yang dapat memperberat penyakit gusi ialah (Things that are not a risk factor for gum disease)…
   a. Karang gigi (Dental calculus)
   b. Tambalan gigi yang masih baik (Proper dental restoration)
   c. Gigi berjejal (Malposition of teeth)

6. Penyakit sistemik yang sering menyebabkan kegoyangan pada gigi adalah (Systemic disease that may contribute to tooth mobility)…
   a. Tipus (Typhoid)
   b. Hipertensi (Hypertension)
   c. Diabetes/penyakit gula (Diabetes Mellitus)

7. Penyakit gusi dapat menjadi semakin parah jika memiliki kebiasaan (Gum disease can be worse due to) …
   a. Makan makanan manis (Sweets)
   b. Minum soda (Soda)
   c. Merokok (Smoking)

8. Kapan Anda memeriksakan gigi secara rutin ke dokter gigi (Time for you to visit a dentist)?
   a. Minimal 6 bulan sekali (At least once in 6 months)
   b. Lebih dari 1 tahun sekali (Once in more than 1 year)
   c. Lebih dari 2 tahun sekali (Once in more than 2 years)

9. Cara yang paling utama dan mudah dilakukan oleh diri sendiri dalam rangka memelihara kesehatan gigi yaitu (Things that can be done individually and improve the oral health)…
   a. Memakai tusuk gigi (toothpick usage)
   b. Menggunakan benang gigi (dental floss usage)
   c. Menyikat gigi 2 kali sehari (Toothbrushing twice a day)

10. Berapa lama sebaiknya total waktu yang dihabiskan saat menyikat gigi (Duration for toothbrushing)?
   a. 30 detik(30 seconds)
   b. 1 menit (1 minute)
   c. 2 menit (2 minutes)

11. Pasien dapat membersihkan plak gigi dengan (Patient can clean dental plaque by) …
   a. Berkumur kencang (hard gargling)
   b. Menyikat gigi (tooth brushing)
   c. Menggunakan tusuk gigi (toothpick usage)

12. Pembersihan karang gigi oleh tenaga medis disebut (Cleaning of the calculus is)...
    a. Desensitasi gigi (Desentization) c. tambal gigi (Dental filling)
    b. Scaling (Scaling)

13. Manfaat menggunakan benang gigi adalah (The propose of dental floss)…
    a. Mengeluarkan makanan yang terselip (Clean the impacted food)
    b. Membersihkan gigi pada area yang tidak tercakup oleh sikat gigi (Clean the area that is missed by conventional toothbrushing)
    c. Membersihkan daerah sela gigi (Clean the interdental area)
    d. Semua benar (All is correct)

14. Bulu sikat gigi yang baik digunakan adalah (The ideal type of bristle)…
    a. Lembut (Soft) c. Kasar (Rough)
    b. Keras (Hard)

15. Salah satu cara mengurangi resiko bau mulut adalah dengan cara (Things to be done to reduce oral malodor) …
    a. Menggunakan tusuk gigi (Toothpick)
    b. Membersihkan lidah dengan alat *tongue scrapper* (Clean the tongue with tongue scrapper)
    c. c. Minum teh manis (Sweet iced tea)

Table S1. Psychomotor Measuring Instrument (checklist of oral hygiene procedures)

No	KEGIATAN (ACTIVITY)	DILAKUKAN (PERFORMED)	TIDAK DILAKUKAN (NOT PERFORMED)
1.	Jumlah pasta gigi yang dikeluarkan dan diberikan pada permukaan sikat gigi terlihat cukup, tidak berlebihan atau kurang, sebanyak setengah dari bulu sikat<br>(The amount of toothpaste given to the toothbrush is enough, not excessive, and not less, more than half of the bristle)		
2.	Menggunakan cermin sebagai alat bantu evaluasi selama proses menyikat gigi<br>(Using the mirror as a tool to evaluate the process of dental cleaning)		
3.	Menggunakan salah satu dari teknik penyikatan gigi dengan benar (teknik sulkular: *bass intrasulcular technique*, *modified bass technique*; teknik sirkular/*scrub*: *fones technique*; teknik vertikal: *roll technique*)<br>(Using one of the following techniques for tooth brushing (Sulcular technique: bass intrasulcular technique, modified bass technique; scrub: fones technique; vertical technique: roll technique)		
4.	Penyikatan dilakukan pada semua permukaan gigi<br>(All dental surface is cleaned)		
5.	Per-sextant dilakukan sejumlah kali gerakan menyikat sesuai dengan teknik yang digunakan<br>(Tooth brushing is performed using a proper technique and done several times)		
6.	Cara memegang tangkai sikat gigi yang benar dan tekanan ringan<br>(Proper hand grip to use the toothbrush and gentle pressure)		
7.	Total waktu penyikatan gigi yang dibutuhkan 1.5 – 2.5 menit<br>(The duration for toothbrushing is 1.5-2.5 mins)		
8.	Menggunakan alat bantu yang disediakan peneliti (*tongue scrapper, dental floss, interdental brush*)<br>(Interdental aids usage (tongue scrapper, dental floss, interdental brush))		
9.	Evaluasi hasil penyikatan gigi dilihat dengan cermin<br>(Mirror is used to evaluate the result of toothbrushing)		
	Jumlah (Total score)		
